# The COVID-19 lockdown: a unique perspective into heterogeneous impacts of transboundary pollution on snow and ice darkening across the Himalayas

**DOI:** 10.1093/pnasnexus/pgad172

**Published:** 2023-06-27

**Authors:** Zhengyang Hou, Yang Li, Liqiang Zhang, Changqing Song, Jintai Lin, Chenghu Zhou, Yuebin Wang, Ying Qu, Xin Yao, Peichao Gao

**Affiliations:** Faculty of Geographical Science, Beijing Normal University, Beijing 100875, China; Faculty of Geographical Science, Beijing Normal University, Beijing 100875, China; Faculty of Geographical Science, Beijing Normal University, Beijing 100875, China; Faculty of Geographical Science, Beijing Normal University, Beijing 100875, China; Laboratory for Climate and Ocean-Atmosphere Studies, Department of Atmospheric and Oceanic Sciences, School of Physics, Peking University, Beijing 100871, China; State Key Laboratory of Resources and Environment Information System, Institute of Geographical Science and Natural Resources, Chinese Academy of Sciences, Beijing 100101, China; Faculty of Geographical Science, Beijing Normal University, Beijing 100875, China; Faculty of Geographical Science, Beijing Normal University, Beijing 100875, China; Faculty of Geographical Science, Beijing Normal University, Beijing 100875, China; Faculty of Geographical Science, Beijing Normal University, Beijing 100875, China

**Keywords:** COVID-19 lockdown, heterogeneous impacts, transboundary pollution, snow and ice darkening, Himalayas

## Abstract

The Tibetan Plateau holds the largest mass of snow and ice outside of the polar regions. The deposition of light-absorbing particles (LAPs) including mineral dust, black carbon and organic carbon and the resulting positive radiative forcing on snow (RFS${}_{\mathrm{LAPs}}$) substantially contributes to glacier retreat. Yet how anthropogenic pollutant emissions affect Himalayan RFS${}_{\mathrm{LAPs}}$ through transboundary transport is currently not well known. The COVID-19 lockdown, resulting in a dramatic decline in human activities, offers a unique test to understand the transboundary mechanisms of RFS${}_{\mathrm{LAPs}}$. This study employs multiple satellite data from the moderate resolution imaging spectroradiometer and ozone monitoring instrument, as well as a coupled atmosphere–chemistry–snow model, to reveal the high spatial heterogeneities in anthropogenic emissions-induced RFS${}_{\mathrm{LAPs}}$ across the Himalaya during the Indian lockdown in 2020. Our results show that the reduced anthropogenic pollutant emissions during the Indian lockdown were responsible for 71.6% of the reduction in RFS${}_{\mathrm{LAPs}}$ on the Himalaya in April 2020 compared to the same period in 2019. The contributions of the Indian lockdown-induced human emission reduction to the RFS${}_{\mathrm{LAPs}}$ decrease in the western, central, and eastern Himalayas were 46.8%, 81.1%, and 110.5%, respectively. The reduced RFS${}_{\mathrm{LAPs}}$ might have led to 27 Mt reduction in ice and snow melt over the Himalaya in April 2020. Our findings allude to the potential for mitigating rapid glacial threats by reducing anthropogenic pollutant emissions from economic activities.

**Significance Statement**

Ice and snow over the Himalayas have been melting at an accelerating, alarming rate in recent decades. Depositions of light-absorbing particles decrease snow albedo and accelerate ice and snow melt. Here, we show that the reductions in human activities during the Indian COVID-19 lockdown and resulting pollution transport have reduced particle depositions and snow melt over the central and eastern Himalayas, while the changes in natural dust transport have been the dominant driver for the western Himalayas. Our findings offer new insights for understanding the radiative balance and water security in the Himalayan region towards establishment of effective protection strategies.

The Tibetan Plateau (TP) holds the largest snow and ice mass outside the polar regions (1, 2), acting as a water storage tower for South and East Asia. Snow and glacial melt in the TP constitute the primary freshwater supply for over 20% of the global population (3). Yet, the glaciers have been melting at an accelerating, alarming rate over the past decade (4, 5), resulting that one-third of Himalayan glaciers inside the TP could be gone by the end of this century due to climate change (6). The glacial retreat of the TP therein further affects the weather, hydrological cycles, and ecosystems at regional and global scales (7). Besides the up to 0.3${}^{\circ }$C per decade of warming on the TP during the past 30 years, previous studies show that deposited light-absorbing particles (LAPs) like dust and black carbon aerosols have significantly contributed to the rapid glacial retreat (8–11).

As an important emitter of LAPs, human activity largely controls the variation of LAP concentrations in ice and snow. Dust aerosols from the Indian Peninsula have been reported to have a strong physical connection with the darkening of snow and ice on the TP (12–14). Thus, assessing the influence of human activities on radiative forcing on snow (RFS${}_{\mathrm{LAPs}}$) is essential for providing valuable guidance for effective mitigation strategies. However, LAP variations reflect a complex mixture of anthropogenic emissions and natural environmental factors and are very sensitive to meteorological conditions. For example, changes in winds can affect LAP transport and deposition, and temperature and snowfall are closely related to the accumulation of LAPs in the snow. Furthermore, the responses of snow and ice radiation forcing to human activities exhibit a heterogeneous spatiotemporal distribution. these factors make estimation of the impacts of human activities on ice and snow melt a challenging task.

The COVID-19 pandemic has created a unique natural experiment for revealing and answering the long-standing question of how reductions in human activities can affect air pollution (15, 16). To cope with the COVID-19 pandemic, the Indian government implemented a national lockdown from 2020 March 25 to 2020 May 31. This led to an unprecedented reduction in economic and transportation activities and pollution levels. Aerosol optical depth (AOD) values dropped sharply and air quality improved across the country (17–19). LAPs concentrations over the Indus Basin have also decreased greatly (15). Such a reduction in LAPs over India might have meant a substantial drop in pollution transported to the TP. In addition, certain amounts of pollutants transported to the Himalayas by the prevailing westerly winds in spring might originate from Nepal and Pakistan as well (1, 20, 21). The two countries implemented lockdowns from March 24 to July 21 and March 23 to August 17 in 2020, respectively. However, the oil/coal CO_2_ emissions of Nepal and Pakistan from 2017 to 2020 were only about 0.5 and 5.4% of the emissions in India, according to Greenhouse Gas Emissions from Energy data. Meanwhile, the burned area from January to May during 2017–2020 were only about 5.5 and 4.8% of those in India, according to the MCD64A1 burned area data (22). The prevalent westerly winds during the lockdown period could easily transport LAPs from northern India to the Himalayas (23). There are more anthropogenic LAPs emissions in northern India compared to southern India (1, 24, 25). Therefore, the anthropogenic LAPs transported to the Himalayas in this season may be mainly tied to emissions in India, and the reduction in human activities during the Indian lockdown period was an important driving factor for reduction in transported pollution. Yet, the specific meteorological conditions also led to significant changes in pollutant concentrations. Therefore, the relationship between LAP reductions on the TP and the decreased human activities in India remain to be addressed.

In this study, using the Indian COVID-19 lockdown as a window, we combine satellite data and model simulations to explore the impacts of large-scale reduction in human activities on RFS${}_{\mathrm{LAPs}}$ along the Himalaya. We quantify the difference between the lockdown period of 2020 and previous years in smoke and dust aerosols on the Indian Peninsula and in the resulting RFS${}_{\mathrm{LAPs}}$ along the Himalaya. We then explore the responses of RFS${}_{\mathrm{LAPs}}$ over the western, central, and eastern Himalayas, respectively, to changes in human activities. The GEOS–Chem chemical transport model and the SNICA (snow ice and aerosol radiation) model [atmosphere–chemistry–snow (GEOS–Chem–SNICAR)] are coupled to simulate the influences of human activities on RFS${}_{\mathrm{LAPs}}$.

To the best of our knowledge, this is the first study that explores the causal mechanism for the impacts of anthropogenic emissions on RFS${}_{\mathrm{LAPs}}$ over the Himalayas. The novelties of this study are as follows. Taking the Indian COVID-19 lockdown as a best-case scenario, we find that anthropogenic emissions and natural environmental factors have different impacts on ice and snow darkening and melt in the three subregions of the Himalaya. We identify the spatial heterogeneity of the impacts, and quantitatively demonstrate their respective contributions to the Himalayan RFS${}_{\mathrm{LAPs}}$. Results of the dynamic mechanisms from the coupled GEOS–Chem–SNICAR model support the findings on the rapid reduction of Himalayan snow and glacial melt in response to major human-made radiative forcing agents. This study provides evidence that reductions in anthropogenic emissions are helpful to decrease snow and glacial melt. Our findings are essential for understanding the radiative balance and water security in the region, and offer valuable guidance for effective mitigation strategies.

## Results

**Fig. 1.**
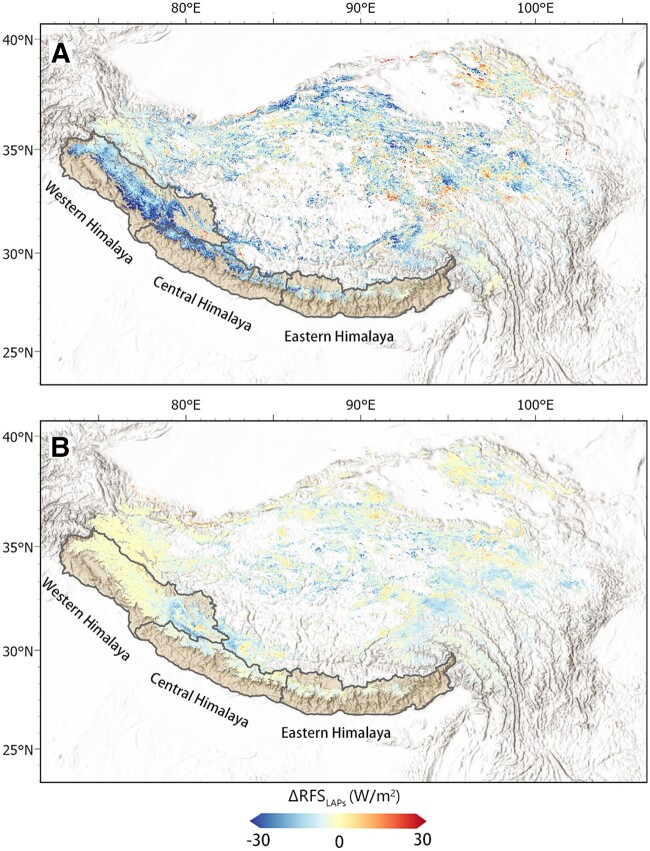
Changes in Himalayan RFS${}_{\mathrm{LAPs}}$ linked to Indian lockdown. Differences between the RFS${}_{\mathrm{LAPs}}$ in 2020 and the average from 2017 to 2019 over the TP. A) Differences during the lockdown period (from April to May). B) Differences during the prelockdown period (from January to March).

The RFS${}_{\mathrm{LAPs}}$ data from MODDRFS retrievals (26), driven by moderate resolution imaging spectroradiometer (MODIS) satellite imagery and the ozone monitoring instrument (OMI) absorbing aerosol optical depth (AAOD) (27) product in January–May from 2017 to 2020 are used to estimate ice and snow pollution and aerosols during the Indian lockdown. The RFS${}_{\mathrm{LAPs}}$ and AAOD data are described in detail in Materials and Methods section. As shown in Fig. 1A, the RFS${}_{\mathrm{LAPs}}$ on the TP, particularly over the Himalayas, had a large decline (by 7.85 W/m^2^ on average) during the Indian lockdown (April–May 2020) compared to the same periods in 2017–2019. Before the lockdown (January–March) in 2020, the RFS${}_{\mathrm{LAPs}}$ values were similar to those in the same periods in 2017–2019, with a difference less than 4 W/m^2^ on average (Fig. 1B).

**Fig. 2.**
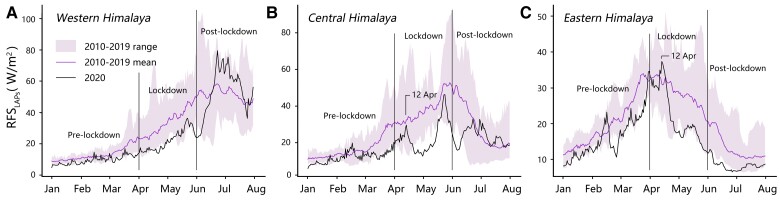
Daily evolution of Himalaya RFS${}_{\mathrm{LAPs}}$. Daily RFS${}_{\mathrm{LAPs}}$ from January to July over the western A), central B), and eastern C) Himalayas. The results are for 2020 and previous years. 2020 April 12 was the date when RFS${}_{\mathrm{LAPs}}$ began to decrease in the central and eastern Himalayas. RFS${}_{\mathrm{LAPs}}$ had no decreasing trend in the western Himalaya.

We also compared the variations of daily average RFS${}_{\mathrm{LAPs}}$ in the western, central, and eastern Himalayas before, during, and after the Indian lockdown in 2020 to the same periods in 2010–2019 (Fig. 2). During the lockdown, RFS${}_{\mathrm{LAPs}}$ in the three regions were at the lowest levels in the recent decade, especially in the western and central Himalayas, with an average decrease of 9.12 and 14.68 W/m^2^, respectively. After the lockdown, RFS${}_{\mathrm{LAPs}}$ on the central and western Himalaya rose rapidly and exceeded the average in 2010–2019, while RFS${}_{\mathrm{LAPs}}$ in the eastern Himalaya gradually returned to the average in 2010–2019.

**Fig. 3.**
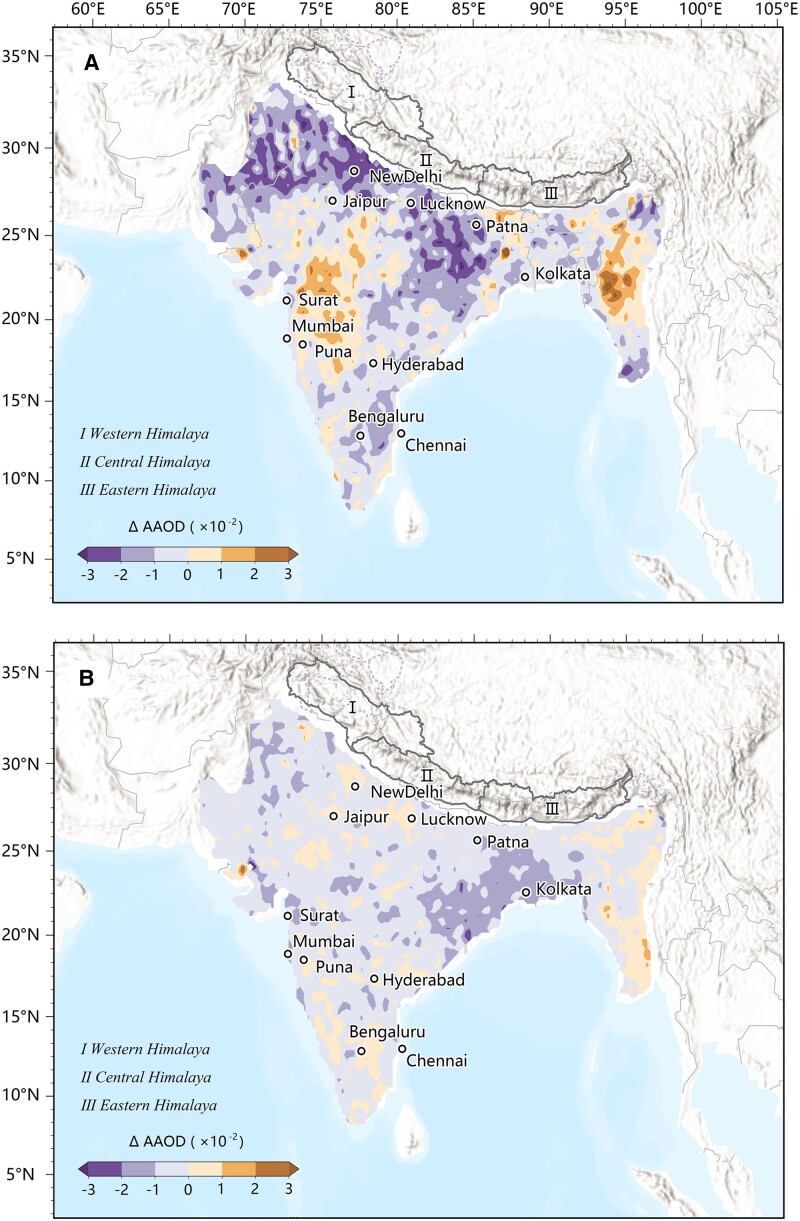
Changes in Indian AAOD linked to lockdown. Differences of the AAOD over Indian Peninsula between 2020 and the same period in previous years (2017–2019). A) Differences during the lockdown (from April to May). B) Differences during the prelockdown period (from January to March).

Previous studies indicated that anthropogenic emissions from South Asia were the main sources of air pollution over the TP (28–30). We examined the changes of air pollutants in South Asia during the lockdown, using AAOD as a dimensionless proxy for the concentration of LAPs in the atmosphere. Over the Indian Peninsula, the AAOD during the lockdown was lower by 13.3% than that in the same period of the previous years (Fig. 3A). The AAOD anomaly in the northern Indian Peninsula near the Himalaya was −20% for the lockdown period (Fig. 3A), compared to the value of −12.1% before the lockdown (Fig. 3B). The day-to-day time series in Online Supplementary Fig. S1 also show the high consistency between AAOD and RFS${}_{\mathrm{LAPs}}$ anomalies in March–May 2020. The association between AAOD and RFS${}_{\mathrm{LAPs}}$ anomalies was less significant in January–February 2020, due to fresh snowfall on snowpack and slow melt over the Himalayas suppressing the accumulation of LAPs on the ice and snow surface. Overall, these results suggest that the anomalies in TP RFS${}_{\mathrm{LAPs}}$ were tied to AAOD over Indian Peninsula. Notably, India is a major emitter of anthropogenic LAPs over the Indian Peninsula (22), but the reduction in AAOD and RFS${}_{\mathrm{LAPs}}$ during the lockdown cannot be entirely explained by a decrease in human activities in India as the meteorological conditions and the nonanthropogenic LAPs coming from multiple countries are also important factors for the changes in AAOD and RFS${}_{\mathrm{LAPs}}$ (31).

We further explored the association of the Indian lockdown with the reduction in RFS${}_{\mathrm{LAPs}}$ over the western, central, and eastern Himalayas, separately. On the western Himalaya, RFS${}_{\mathrm{LAPs}}$ had a large decline during the lockdown period compared to the same period in previous years (Fig. 2A). This, however, might not have been triggered solely by the reduction in transboundary anthropogenic pollution for several reasons. First, the reduction in RFS${}_{\mathrm{LAPs}}$ for the same period from 2018 to 2019 (with an average of 11.31 W/m^2^) (Online Supplementary Fig. S2B) was greater than from 2019 to 2020 (9.12 W/m^2^). Second, the positive anomaly in the RFS${}_{\mathrm{LAPs}}$ during the post-lockdown period (Fig. 2A) was in contrast to the continued decline in Indian emissions of CO_2_ (Online Supplementary Fig. S4), which was used here as a proxy of anthropogenic pollution due to the lack of pollutant emission data. Therefore, the RFS${}_{\mathrm{LAPs}}$ reduction over the western Himalaya during the lockdown period might also have been contributed by natural environmental changes.

**Fig. 4.**
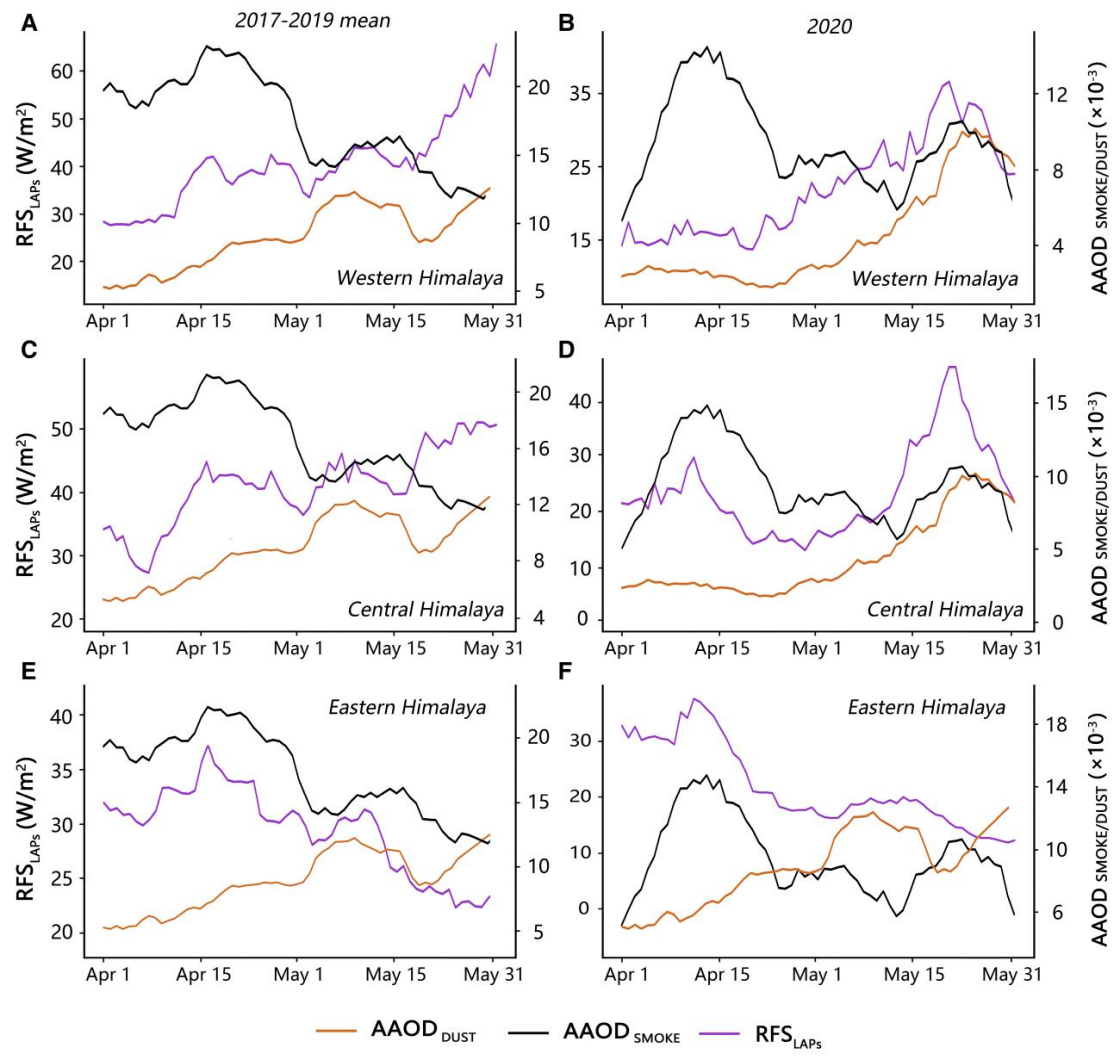
Daily changes in aerosols and RFS${}_{\mathrm{LAPs}}$ from April to May. Daily AAOD_SMOKE_/AAOD_DUST_ in Indian peninsula and RFS_LAPs_ over the western A-B), central C-D), and eastern E-F) Himalayas. The left panels A, C, E) and right panels B, D, F) respectively show the changes of both aerosols and RFS_LAPs_ in April-May 2020 and the same periods in 2010–2019.

Dust emitted from deserts is an important natural source of LAPs (32). The dust-induced RFS${}_{\mathrm{LAPs}}$ is predominantly dominated by natural environmental factors like wind, humidity, and precipitation. During the premonsoon period from mid-March to May before the onset of the South Asian summer monsoon (33–35), dust storms are frequently carried to the Himalayas from the upwind arid regions at about 80${}^{\circ }$E, including Saudi Arabia, Pakistan, Thar Desert and Sahara. (36, 37). A large amount of dust aerosols are transported into the western Himalaya by the prevailing westerly wind (38) (Online Supplementary Fig. S3) (Materials and Methods section). Dust aerosols increased slightly from 2017 to 2018, and decreased from 2018 to 2020 over the northern India (Online Supplementary Fig. S3), consistent with the interannual variation of RFS${}_{\mathrm{LAPs}}$ on the western Himalaya. Furthermore, the correlation coefficient for day-to-day variation between RFS${}_{\mathrm{LAPs}}$ and AAOD of dust aerosols (AAOD${}_{\mathrm{DUST}}$) was 0.86 during the Indian national lockdown period and was 0.74 in the same period in 2017–2019 (Fig. 4A and B). In contrast, the day-to-day variation of RFS${}_{\mathrm{LAPs}}$ on the western Himalaya was not correlated with AAOD of smoke aerosols (AAOD${}_{\mathrm{SMOKE}}$) from April to May in 2017–2020. These results suggest that the decline in RFS${}_{\mathrm{LAPs}}$ on the western Himalaya during the Indian lockdown period in 2020 was mainly contributed by the reduction in transported desert dusts.

The central and eastern Himalayas are close to the hotspots of Indian anthropogenic pollution (Online Supplementary Fig. S3). Available observations have shown that large amounts of polluting aerosols accumulate in the central and eastern Himalayas (39). During the premonsoon periods, these LAPs are carried by southwesterly wind to the higher altitudes to be deposited to snow/ice over the central and eastern Himalayas (13, 31, 40).

**Fig. 5.**
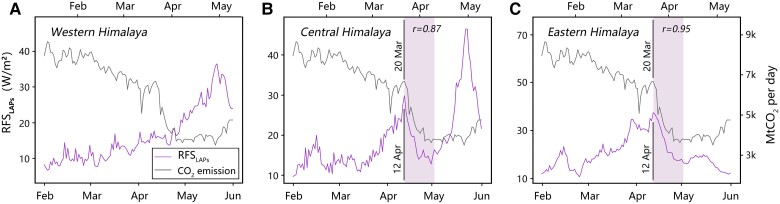
Daily changes in CO_2_ emission and RFS${}_{\mathrm{LAPs}}$ from February to May 2020. A–C) in the western Himalaya, central Himalaya and eastern Himalaya, respectively. 2020 April 12 was the date when RFS${}_{\mathrm{LAPs}}$ began to decrease in the central and eastern Himalayas. 2020 March 20 was the date when the CO_2_ emission began to decrease. The time-lagged correlation coefficients are for CO_2_ emission and RFS${}_{\mathrm{LAPs}}$ over the 20 days after their descent dates (April 12–May 1 for RFS${}_{\mathrm{LAPs}}$ and March 20–April 8 for CO_2_ emission).

On the eastern Himalayas, the RFS${}_{\mathrm{LAPs}}$ was on the rise from 2017 to 2019 (Online Supplementary Fig. S2G and H), different from the interannual variations in mineral dusts (Online Supplementary Fig. S3A–C). Anthropogenic CO_2_ emissions, as a proxy to air pollution, plummeted by nearly 50% from 2020 March 20 to 2020 April 8 compared to the same period in 2019 in India (Online Supplementary Fig. S4). Likely as a lagged effect, the RFS${}_{\mathrm{LAPs}}$ had a substantial decrease on the eastern Himalayas from April 12 to May 1. The lagged correlation for day-to-day variations between CO_2_ emissions and RFS${}_{\mathrm{LAPs}}$ on the eastern Himalaya reached 0.95 during 20 days since their descents (March 20–April 8 for CO_2_ vs April 12–May 1 for RFS${}_{\mathrm{LAPs}}$) (Fig. 5B). In contrast, there was a consistent increase of RFS${}_{\mathrm{LAPs}}$ on the western Himalaya from January to May (Fig. 5A). Furthermore, the day-to-day correlation between RFS${}_{\mathrm{LAPs}}$ over the eastern Himalayas and smoke aerosols was much higher than that between RFS${}_{\mathrm{LAPs}}$ and dust aerosols (Fig. 4E and F). The results suggest that the RFS${}_{\mathrm{LAPs}}$ over the eastern Himalayas was more sensitive to anthropogenic pollution than to natural factors during the Indian lockdown.

Dust aerosols transported to the western Himalaya could be further carried to the central Himalaya (41). Meanwhile, the central Himalaya could be affected by a large amount of atmospheric black carbon (BC) dominated by anthropogenic sources (21, 42). During the Indian lockdown, on the one hand, RFS${}_{\mathrm{LAPs}}$ in the central Himalaya and AAOD${}_{\mathrm{DUST}}$ in India showed a high correlation between 0.77 during the lockdown in 2020 and 0.72 for the same period in 2017–2019 (Fig. 4). On the other hand, the RFS${}_{\mathrm{LAPs}}$ on the central Himalaya had a large reduction from April 12 to May 1, similar to the eastern Himalaya and opposite to the western Himalaya. The lagged day-to-day correlation between the RFS${}_{\mathrm{LAPs}}$ over the central Himalaya and CO_2_ emissions in India reached 0.87 during 20 days since their descents (March 20–April 8 for CO_2_ vs April 12–May 1 for RFS${}_{\mathrm{LAPs}}$) (Fig. 5). We conclude that RFS${}_{\mathrm{LAPs}}$ on the central Himalaya was influenced by both natural factors and anthropogenic emissions.

## Mechanisms of heterogeneous impacts across the Himalayas

We used the GEOS–Chem–SNICAR model to further investigate the relative contributions of anthropogenic emissions and natural environmental factors to RFS${}_{\mathrm{LAPs}}$ across the Himalayas during the Indian lockdown in three scenarios (Materials and Methods section). In Scenario 1, we used the meteorology in 2020, and set a 50% emissions reduction in India during the lockdown. Since the anthropogenic pollutant emission inventory in 2020 were not available, a 50% reduction was used to be consistent with the Indian CO_2_ emission reduction in April 2020 compared to the same period in 2019 (43) (Online Supplementary Fig. S4). In Scenario 2, we used the meteorology in 2020 but did not change the emissions, representing the counterfactual scenario with respect to the lockdown. In Scenario 3, we used the meteorology in 2019 and kept the emissions unchanged.

**Fig. 6.**
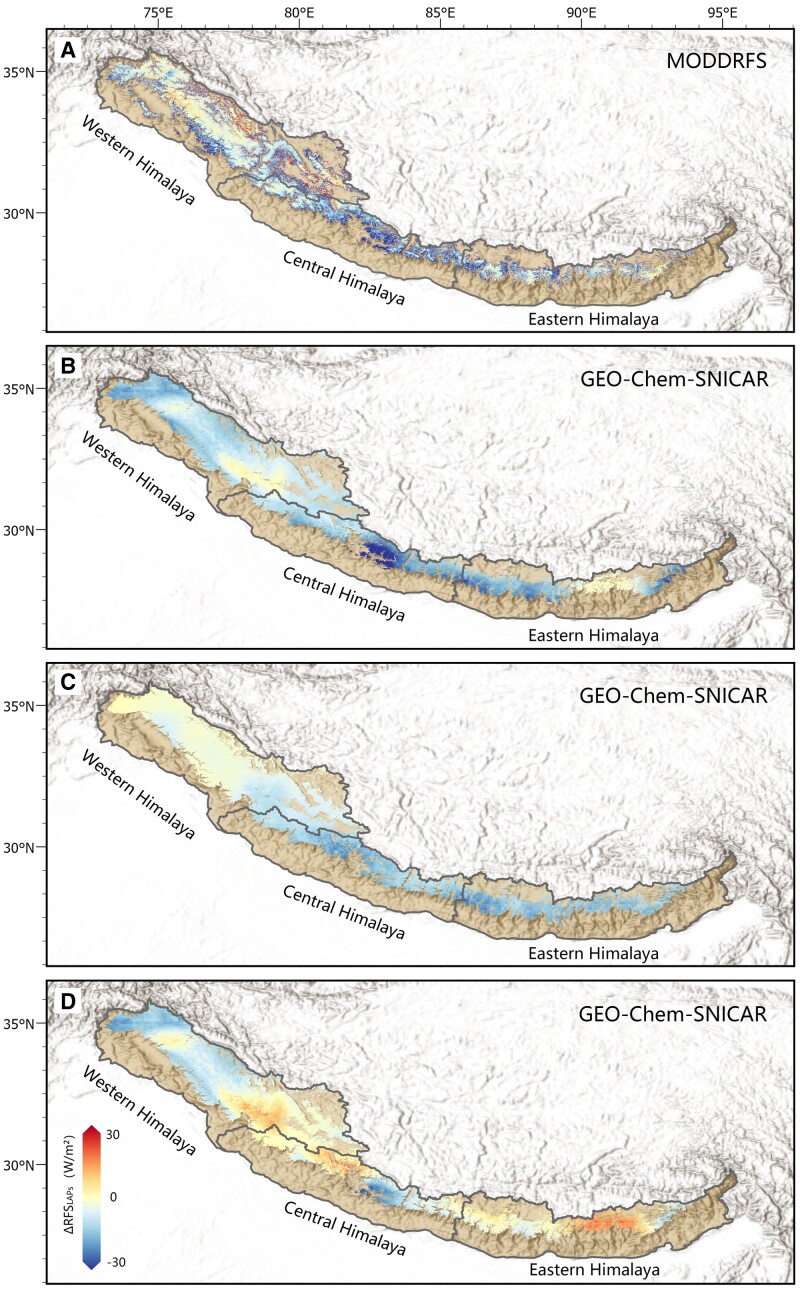
Differences of RFS${}_{\mathrm{LAPs}}$ in the three scenarios over the Himalaya. A) Differences between April 2020 and April 2019 from the MODDRFS-RFS${}_{\mathrm{LAPs}}$ dataset. B) Difference between Scenarios 1 and 3. C) Differences between Scenarios 1 and 2. D) Differences between Scenarios 2 and 3.

The difference between Scenarios 1 and 3 represents the variation in RFS${}_{\mathrm{LAPs}}$ driven by interannual changes in meteorology and emission together, as occurring in reality. As illustrated in Fig. 6A, the simulated difference in RFS${}_{\mathrm{LAPs}}$ (Fig. 6B) was consistent with the MODDRFS-RFS${}_{\mathrm{LAPs}}$, i.e. 9.36, 16.35, and 13.34 W/m^2^ (the simulated difference) vs 8.87, 16.04, and 11.06 W/m^2^ (the MODDRFS-RFS${}_{\mathrm{LAPs}}$) on western, central, and eastern Himalayas (Online Supplementary Table S1), indicating the capability of the GEOS–Chem–SNICAR model to capture the effects of meteorology and emissions on RFS${}_{\mathrm{LAPs}}$.

The difference between Scenarios 1 and 2 (Fig. 6C) represents the effect of Indian emission reduction alone. The RFS${}_{\mathrm{LAPs}}$ results reveal that the emission impacts have large spatial heterogeneity across the Himalaya. As listed in Online Supplementary Table S1, under a 50% reduction in anthropogenic emissions, the RFS${}_{\mathrm{LAPs}}$ on the eastern and central Himalayas decreased (by 14.68 and 13.26 W/m^2^) more than those on the western Himalaya (by 4.39 W/m^2^) (by 4.39 W/m^2^), indicating that human activities have played a greater role in decrease of RFS${}_{\mathrm{LAPs}}$ (the difference between Scenarios 1 and 2) in eastern and central Himalayas than western, with the contribution by 110.5% and 81.1 vs 46.8%.

The difference between Scenarios 2 and 3 represents the sole effect of the natural environmental factors on RFS${}_{\mathrm{LAPs}}$. Fig. 6D shows that RFS${}_{\mathrm{LAPs}}$ over the eastern Himalaya during the Indian lockdown was higher than those in 2019 (by 1.34 W/m^2^ on average). In contrast, there were apparent reductions over the western and central Himalayas (by 4.97 and 3.09 W/m^2^, respectively), revealing that RFS${}_{\mathrm{LAPs}}$ over the western Himalaya was more sensitive to the natural variability, contributed by 53.2% to decrease of RFS${}_{\mathrm{LAPs}}$ (the difference between Scenarios 1 and 2) vs 18.9 and −10.5% in central and eastern Himalayas. Overall, these modeling results indicate large reductions in anthropogenic emissions were not the only factor for the decrease of RFS${}_{\mathrm{LAPs}}$ over the Himalaya during the Indian lockdown, and were chiefly responsible for 71.6%, consistent with the independent, observation-based analysis.

We further converted the simulated RFS${}_{\mathrm{LAPs}}$ under the three scenarios into ice and snow melt to estimate the effect of the reduced anthropogenic emissions during the Indian lockdown in 2020 (see Materials and Methods section). Compared with the same period in 2019, ice and snow melt was declined by 27.49 Mt (70.7%) over the Himalayas in April 2020 due to reduced anthropogenic emissions, while the reduction in snowmelt caused by the natural factors was 11.44 Mt (29.3%) (Online Supplementary Table S2). The much larger effect of anthropogenic emissions on ice and snow melt over the Himalayas suggests a great potential of cutting anthropogenic emissions to curb the rapid snow and glacial melt.

## Disscusion

This study has employed multiple satellite-based data and an GEOS–Chem–SNICA model to assess the substantial changes in RFS${}_{\mathrm{LAPs}}$ across the Himalaya caused by transboundary anthropogenic pollution and natural environmental factors during the Indian COVID-19 lockdown. Heterogeneous spatial patterns exist in the LAPs-induced snow/ice darkening. Desert dust particles dominate the ice and snow darkening on western Himalaya. The LAPs-induced darkening has larger responses to transboundary anthropogenic pollution on central and eastern Himalayas than on the western Himalaya.

Over the recent years, due to reduction in mineral dust emissions in Central Asia, fewer dusts might have been deposited on ice and snow of the Himalaya, leading to less dust-induced glacial and snow melt (44, 45). In contrast, more BC might have been deposited because of increasing anthropogenic emissions in South Asia (46). Furthermore, for a given amount of mass, BC has greater impacts on ice and snow melt than dust due to its larger mass absorption efficiency (31). These factors drive the spatial disparities in ice and snow melt across the Himalaya. And our results further show how short-term pollution disruptions can have a substantial additional effect.

This study offers evidence that the reduction in transboundary LAPs has a remarkable beneficial effect on the reduction of the Himalayan snow and ice melt. It provides an opportunity for targeted emission mitigation to constrain the timing and magnitude of future glacier retreat. Mineral dust transported from the Thar Desert by westerly winds deeply influences the RFS${}_{\mathrm{LAPs}}$ and the rate of ice and snow melting in the western and central Himalayas. Thus reduction in deforestation and overexploitation of the Indus River Basin (47–49) may help improve the ecological environments around the Thar Desert, and further decrease mineral dust transport to melt ice and snow on the TP. Furthermore, continued use of fossil fuels in the future would increase ice and snow melt by increasing the LAPs, in addition to leading to sustained global warming (8). If present-day emission of greenhouse gases continue, about 60–70% of the glacier over the Himalayas would be lost in this century (50, 51), affecting fresh water supplies for billions of people. Thus reduction in fossil fuels in the Indian Peninsula could substantially alleviate warming and ice/snow melts on the TP and protect its precious water resource.

This study analyzes the response of RFS${}_{\mathrm{LAPs}}$ over the Himalaya to Indian anthropogenic emissions as a window to analyze the effect of the COVID-19 lockdown, while the effects of pollution transported from other regions (e.g. Southeast Asia and China) were not included. The changes in RFS${}_{\mathrm{LAPs}}$ in other seasons were not explored in detail. A comprehensive analysis of RFS${}_{\mathrm{LAPs}}$ in all months as well as the transboundary pollution from global emissions will offer a complete picture of how human activities are linked to the TP snow/ice darkening and melting through light-absorbing aerosols.

## Materials and methods

### RFS${}_{LAPs}$

The RFS${}_{\mathrm{LAPs}}$ data were taken from the Jet Propulsion Laboratory (https://snow.jpl.nasa.gov/portal/data/). The data were derived from the surface reflectance data (MOD09GA and MYD09GA) (52) from NASA’s MODIS based on the Snow-Covered Area and Grain size (MODSCAG) model (53) and the Dust Radiative Forcing in Snow (MODDRFS) model (26). The MODSCAG model used multiple endmembers linear spectral mixture analysis, the Mie theory and a discrete-ordinates radiative transfer model to estimate subpixel snow-covered area and snow albedo. Snow grain radii were estimated by the normalized snow grain size indices in MODSCAG. The MODDRFS determined the spectral reflectance differences between the measured MODIS spectrum and the modeled clean snow spectrum with the same snow grain radius. Integration of the bandwise multiplication of the spectral difference with near-infrared spectral irradiance (0.350–0.876 μm) that accounts for terrain variations gives the instantaneous surface radiative forcing. The retrieved radiative forcing from MODIS is instantaneous value rather than daily average. The RFS${}_{\mathrm{LAPs}}$ data were validated with over 6 years of radiometer measurements (54). Here, we took the JPL RFS${}_{\mathrm{LAPs}}$ data during the 2010–2020 period and carried out 7-day linear interpolation pixel by pixel to further improve the integrity over time and space.

### Absorbing aerosol optical depth

AAOD and aerosol type data were taken from the OMI level 2 near-UV aerosol products (http://disc.gsfc.nasa.gov). OMI is a nadir-viewing near-UV/Visible charge coupled device spectrometer aboard the Aura satellite. OMI measurements cover a spectral region of 264–504 nm. Because of the large sensitivity of the OMI near-UV observations to particle absorption, AAOD is a reliable quantitative aerosol parameter (55). Many studies used the data product to identify absorbing aerosols from biomass burning and desert dusts (56, 57). We used the OMI Level-2 AAOD data to represent the absorbing aerosols over the Indian Peninsula.

The OMI aerosol products were generated by the OMI/Aura near-ultraviolet aerosol retrieval algorithm (OMAERUV) (55) which used a set of aerosol models to account for the presence of carbonaceous aerosols from biomass burning, desert dust, and sulfate-based aerosols. AAODs at 354, 388, and 500 nm were obtained from OMI aerosol products. AAOD at 388 nm was derived directly from the radiance observations and are chosen here. AAOD at 354 and 500 nm were converted from 388 nm, and considered less reliable because the transformation relied on the spectral dependence of the aerosol models assumed in the algorithm. The OMAERUV algorithm used the Lambert equivalent reflectivity data which were only applied to the regions with lower than 600 hPa. Therefore, AAOD data are missing over the Himalaya.

Aerosols are classified into three types: dust, smoke, and sulfate. Aerosol type determination is carried out on the basis of the magnitudes of the near-ultraviolet aerosol index and carbon monoxide index (COI) parameters. In using the COI, the effect of background upper tropospheric carbon monoxide, which might not be necessarily associated with local emissions of carbonaceous aerosols, was removed (24, 52). The smoke aerosols were mainly produced by biomass burning, containing dominant carbonaceous compounds such as black carbon and organic carbon, while the dust aerosols were produced from arid and semiarid areas (such as deserts) under conducive meteorological conditions (27, 58).

The pixel size for AAOD was 13×24km at nadir and 28×150km at the swath edges with an exact 16-day repeat cycle. The AAOD data were resampled to the resolution of $0.25^{\circ }\times 0.25^{\circ }$, and the data of the previous 7 days and the next 8 days were used to synthesize the data for the current date.

The daily AAOD for each aerosol type (AAOD${}_{\mathrm{DUST}}$ and AAOD${}_{\mathrm{SMOKE}}$) was calculated based on AAOD and aerosol type from OMI aerosol products. Each pixel provides both an AAOD value and an aerosol type. We made a mask to set the AAOD values at pixels corresponding to nondust aerosol types to 0, and took the average of AAOD in the Indian peninsula as AAOD${}_{\mathrm{DUST}}$. AAOD${}_{\mathrm{SMOKE}}$ was obtained in a similar way.

### CO_2_ emission data

The oil/coal CO_2_ emission statistics for India, Pakistan, and Nepal from 2017 to 2019 were based on the Greenhouse Gas Emissions from Energy database. This database includes annual CO_2_ emissions from fuel combustion and fugitive emissions in 203 countries worldwide. We only considered CO_2_ emissions from the combustion of oil and coal, and excluded relatively cleaner sources of energy such as gas, which produce fewer LAPs. The data are freely available from the International Energy Agency (https://www.iea.org/).

The daily CO_2_ emission data were derived from Carbon Monitor (43), which tracked the changes in CO_2_ emissions from fossil fuel combustion and cement production since 2019 January 2019. Daily CO_2_ emission values were constructed based on activities from power generation, industry, road transportation, aviation and maritime transportation, as well as commercial and residential sectors. The Carbon Monitor dataset shows the variations in CO_2_ emissions influenced by weekends and holidays as well as the COVID-19 pandemic (43). The data can be obtained from https://www.carbonmonitor.org.cn/.

### Burned area data

We used MCD64A1.006 burned area data (22) to estimate the burned area in India, Nepal, and Pakistan from January to May for the years 2017 to 2020. The MCD64A1 burned area data is a remote sensing data product obtained from the MODIS satellite. The MCD64A1 data product contains global fire information, providing spatial distribution and time-series data of global fires with a resolution of 500 m for each pixel. The MCD64A1 data product is freely available from https://earthdata.nasa.gov/.

### Wind data

Wind data were taken from the Fifth Generation ECMWF Reanalysis (ERA5) of the global climate and weather. The wind vectors are at $0.25^{\circ }\times 0.25^{\circ }$ resolution and averaged from April to May in 2020 at 500 hPa. The data are freely available from the Climate Data Store (https://cds.climate.copernicus.eu/).

### GEOS–Chem–SNICAR simulations

GEOS–Chem is a 3D chemical transport model for simulations of atmospheric compositions on local to global scales. We used GEOS–Chem version 13.2.0 nested over Asia (60${}^{\circ }$E–145${}^{\circ }$E, 0${}^{\circ }$N–60${}^{\circ }$N) at a $0.5^{\circ }\times 0.625^{\circ }$ horizontal resolution with 47 vertical levels. We employed The MERRA-2 assimilated meteorological data to drive the simulations. The nonlocal scheme implemented by Lin and McElroy (59) was adopted for Boundary layer mixing. GEOS–Chem simulated detailed tropospheric oxidant-aerosol chemistry, including dry deposition (60–63) and wet deposition (64–66) of gases and particles. The simulation of aerosols included sulfate, nitrate, ammonium, primary and secondary organic aerosols, BC, natural dusts in four advected size ranges, and sea salts. Global anthropogenic emissions follow the Community Emissions Data System (CEDS) inventory (67). For Asia, the MIX regional inventory (v2015-6) was utilized for anthropogenic emissions. Natural emissions from lightning, vegetation, seabirds, and volcanoes were also included in this model. All emissions were managed by the Harvard–NASA Emissions Component (HEMCO) module (68). We ran the model from 1 March to 30 April in 2019 and 2020. The simulation for March was used for spin-up, and that in April for analysis.

The snow, ice, and aerosol radiation (SNICAR) model employed the snow albedo theory (parameterization) based on Warren and Wiscombe (69) and the two-stream radiative approximation for multiple layers (70). SNICAR simulated the albedo and radiation absorption of snow and the radiation effect of aerosol components in each layer of snow by using ice refraction index data from Picard (71) and optical characteristics of dusts from the Sahara Desert (72), with the reflectivity of the underlying surface set at 0.25.

In the GEOS–Chem–SNICAR model, BC and dust deposition on ice and snow were calculated through dry and wet deposition processes. The SNICAR and GEOS–Chem simulate four tracers of dust at different size bins. We established a mapping relationship between these tracers in the two models (Online Supplementary Table S3). SNICAR model contains two kinds of BC (externally and internally mixed) to present the enhancement of light absorption by snow particles containing black carbon. These two kinds of BC tracers correspond to the dry deposition and wet deposition mass of BC in GEOS–Chem, respectively. Considering the influence of snow cover fraction in the simulation process, the pollutant deposition fluxes simulated by the GEOS–Chem model have been multiplied by snow cover fraction before use. The snow cover fraction data were from MODSCAG retrievals.

In the scenario simulating the impact of human activities on snow pollution, we assumed that snow grain radii and thickness were not affected by anthropogenic pollution. The snow grain radii data in GEOS–Chem–SNICAR were from the MODSCAG retrievals, and the average absolute error of the snow grain radii in MODSCAG was 51 μm based on field measurements (53). Since the minimum snow grain radius allowed in SNICAR was 30 μm, the snow grain radii below 30 μm were set to 30 μm. The snow density was considered to be a constant value of 150 kg/m${}^{3}$. The snow thickness data were taken from the monthly synthetic $0.5^{\circ }\times 0.625^{\circ }$ MERRA-2 reanalysis data (73). The data are freely available from the Goddard Earth Sciences Data and Information Services Center (https://disc.gsfc.n-asa.gov/). In the regions with snow thickness less than 3 cm, the LAPs were considered to be fully integrated into the ground layer, so RFS${}_{\mathrm{LAPs}}$ was not calculated in these regions. The solar radiation flux data were from a global high-resolution (3 h, 10 km) surface solar radiation dataset (74). We used the data at 12:00 local time every day in April 2018 and synthesized the April mean flux. The data are freely available from the National Tibetan Plateau Third Pole Environment Data Center (https://data.tpdc.ac.cn/zh-hans/data/). The Cubic spline interpolation was used for resampling of the snow thickness and solar radiation flux to a 500 m resolution.

### Snowmelt estimates

Snowpack in grid cells with temperatures $>0^{\circ }$C was considered to be melting. Snowmelt was calculated as follows:


(1) $$Snowmelt=RFS_{LAPs}\times A\times g\times \Delta t\sum_{t}\frac{SW_{t}\times MTgt0_{t}}{{\bar{SW}}_{12}}$$

where *A* is the snow-cover area; *g* is enthalpy of fusion of water (334 J g${}^{-1}$); Δt is the temporal resolution of the radiation flux data (3 h); $SW_{t}$ is the radiation flux at local time *t* of each day; ${\bar{SW}}_{12}$ is the mean radiation flux at 12:00 local time of each day; and $MTgt0_{t}$ is a dummy variable of melting (0 when temperatures $>0^{\circ }$C and 1 otherwise). The range of *t* is from 0:00 on April 1 to 24:00 on April 30, with an interval of 3 h. The RFS${}_{\mathrm{LAPs}}$ was simulated by the GEOS–Chem–SNICAR model. To get instantaneous values of RFS${}_{\mathrm{LAPs}}$ at the same time period as the MODDRFS result, the mean solar radiation flux at 12:00 local time every day ${\bar{SW}}_{12}$ was used as input in the simulation. Therefore, the ratio of $SW_{t}$ to ${\bar{SW}}_{12}$ can be taken as the weight of RFS${}_{\mathrm{LAPs}}$ every 3 h.

## Supplementary Material

pgad172_Supplementary_Data

## Data Availability

RFS${}_{\mathrm{LAPs}}$ data are available from https://snow.jpl.nasa.gov/portal/data/. The AAOD dataset used in this study can be downloaded from http://disc.gsfc.nasa.gov. The International Energy Agency oil/coal CO_2_ emission dataset used in this study can be downloaded from https://www.iea.org/. The daily CO_2_ emission data used in this study can be downloaded from https://www.carbonmonitor.org.cn/. The MCD64A1.006 burned area data used in this study can be downloaded from https://earthdata.nasa.gov/. Wind data used in this study can be downloaded from https://cds.climate.copernicus.eu/. The MERRA-2 reanalysis data used in this study can be downloaded from https://disc.gsfc.n-asa.gov/. The solar radiation flux data used in this study can be downloaded from https://data.tpdc.ac.cn/zh-hans/data/. All data are included in the manuscript and/or Online Supplementary material.
