# Supplementary material for: The COVID-19 lockdown: a unique perspective into heterogeneous impacts of transboundary pollution on snow and ice darkening across the Himalayas
Source: PNAS Nexus. 2023 Jun 27;2(6):pgad172. doi: 10.1093/pnasnexus/pgad172 (PMC10299077; doi:10.1093/pnasnexus/pgad172)
Supplement: pgad172_Supplementary_Data [file pgad172_supplementary_data.zip › PNASNEXUS-PNASNEXUS-2022-00897-s02.pdf]

# Supporting Information for

## The COVID-19 lockdown: A unique perspective into heterogeneous impacts of transboundary pollution on snow and ice darkening across the Himalayas

**Zhengyang Hou, Yang Li, Liqiang Zhang, Changqing Song, Jintai Lin, Chenghu Zhou, Yuebin Wang, Ying Qu, Xin Yao, Peichao Gao**

**Liqiang Zhang.**
**E-mail: zhanglq@bun.edu.cn**

**This PDF file includes:**

Figs. S1 to S4
Tables S1 to S3

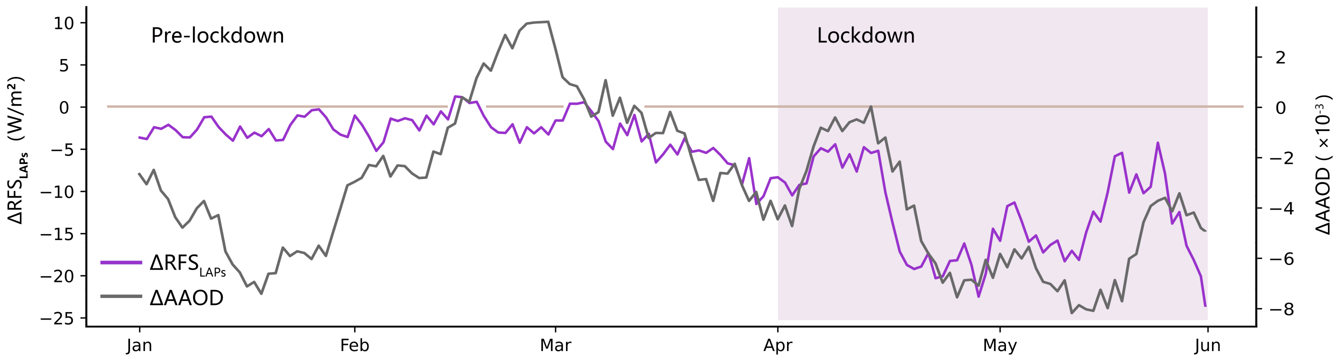


**Fig. S1.** Daily evolution of Himalaya $RFS_{LAPs}$ linked to Indian AAOD. Differences between 2020 and previous years (2017–2019) in daily AAOD over Indian Peninsula and in daily $RFS_{LAPs}$ over the Himalayas.

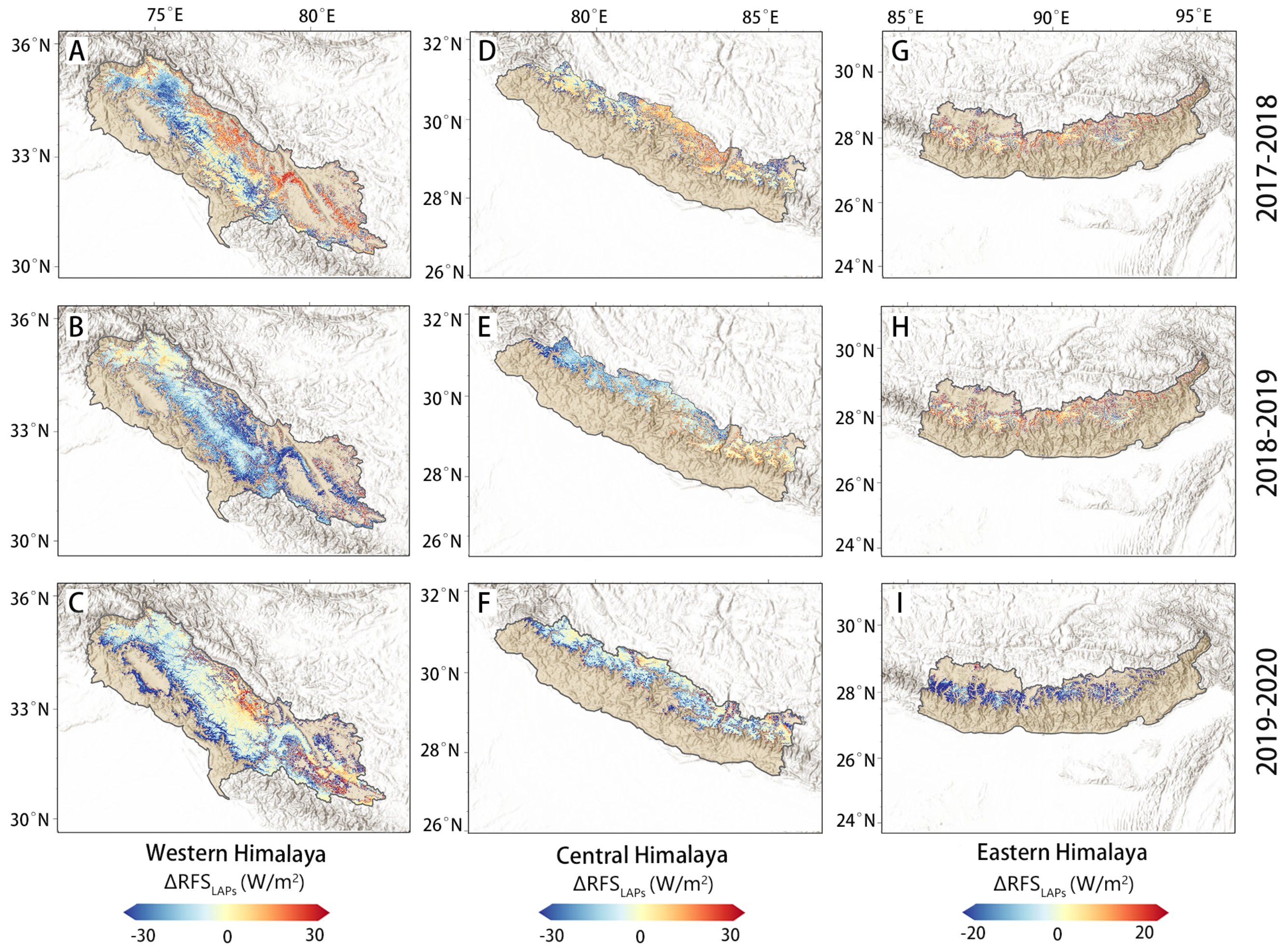


**Fig. S2.** Interannual changes in Himalaya $RFS_{LAPs}$ in April and May. A, B and C show the year-on-year differences in 2018-2017, 2019-2018, and 2020-2019 on the western Himalaya. D, E and F are for the central Himalaya; G, H and I are for the eastern Himalaya.

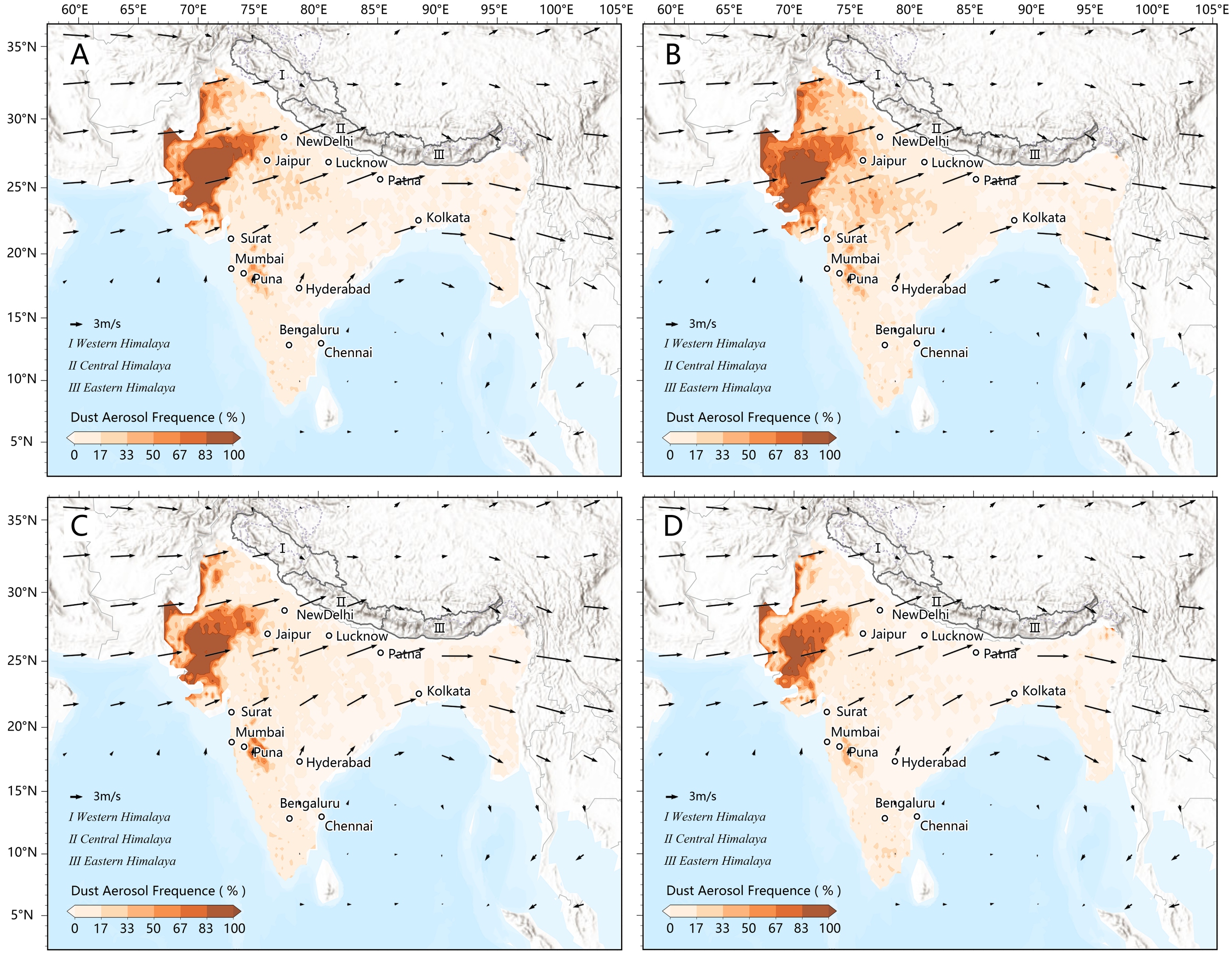


**Fig. S3.** Distributions of mineral dust aerosols over the Indian Peninsula in April and May from 2017 to 2020. A-D represent the years from 2017 to 2020, respectively. The percent values in the figures denote the days with dust aerosols that occurred in April-May divided by the total number of days in the two months. The black vectors denote winds at 500 hPa from the ERA5-reanalysis dataset.

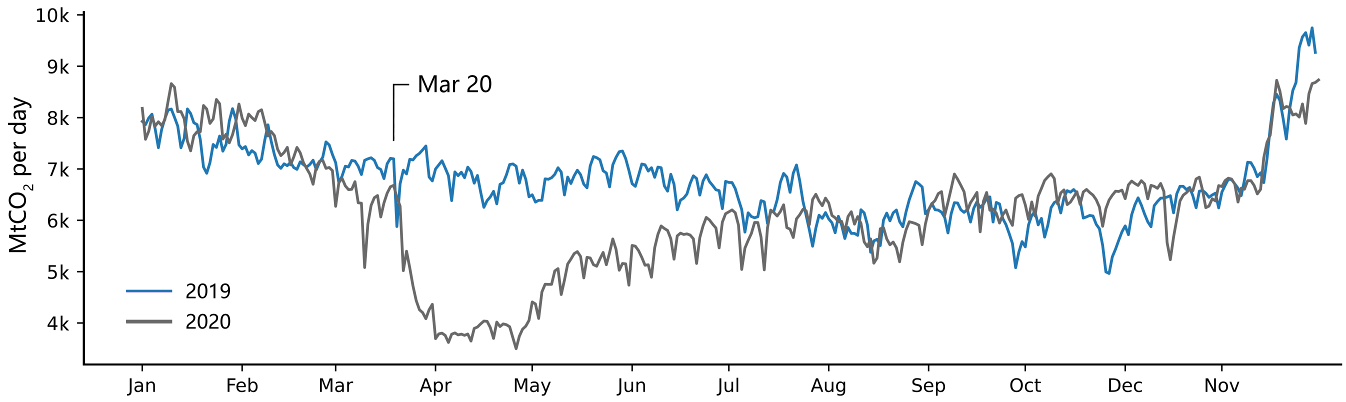


**Fig. S4.** Daily $CO_2$ emissions in India in 2019 and 2020 ().

**Table S1. Contributions of anthropogenic emissions and natural environment factors to $RFS_{LAPs}$ in over the Himalayas in 2020 (unit: $W/m^2$)**

| | Western Himalaya | Central Himalaya | Eastern Himalaya | the Himalaya |
|---|---|---|---|---|
| Anthropogenic emissions | -4.39 | -13.26 | -14.68 | -8.32 |
| Natural environment factors | -4.97 | -3.09 | 1.34 | -3.33 |
| All | -9.36 | -16.35 | -13.34 | -11.65 |
| MODDRFS | -8.87 | -16.04 | -11.71 | -11.01 |

**Table S2. Contributions of anthropogenic emissions and natural environment factors to ice and snow melt in over the Himalayas in 2020 (unit: Mt)**

| | Western Himalaya | Central Himalaya | Eastern Himalaya | the Himalaya |
|---|---|---|---|---|
| Anthropogenic emissions | -2.96 | -10.15 | -15.50 | -27.49 |
| Natural environment factors | -6.90 | -4.79 | 1.88 | -11.44 |
| All | -9.86 | -14.94 | -13.62 | -38.93 |

**Table S3. Dust size mapping between SNICAR and GEOS-Chem**

| | | SNICAR | | | |
|---|---|---|---|---|---|
| | | 0.1-1$\mu$m | 1-2.5$\mu$m | 2.5-5$\mu$m | 5-10$\mu$m |
| GEOS-Chem | 0.7$\mu$m | 1 | 0 | 0 | 0 |
| | 1.4$\mu$m | 0 | 1 | 0 | 0 |
| | 2.4$\mu$m | 0 | 0.5 | 0.5 | 0 |
| | 4.5$\mu$m | 0 | 0 | 0.5 | 0.5 |
